# *H2BFWT* Variations in Sperm DNA and Its Correlation to Pregnancy

**DOI:** 10.3390/ijms25116048

**Published:** 2024-05-31

**Authors:** Houda Amor, Ingolf Juhasz-Böss, Riffat Bibi, Mohamad Eid Hammadeh, Peter Michael Jankowski

**Affiliations:** 1 Departement of Obstetrics and Gynecology, IVF Laboratory, Saarland University Clinic, 66421 Homburg, Germany; mehammadeh@yahoo.de (M.E.H.);; 2 Departement of Obstertics and Gynecology, IVF Laboratory, Freiburg University Clinic, 79106 Freiburg, Germany; 3 Department of Animal Sciences, Faculty of Biological Sciences, Quaid-i-Azam University Islamabad, Islamabad 45320, Pakistan

**Keywords:** male infertility, sperm DNA, SNPs, *H2BFWT*, pregnancy, ICSI

## Abstract

Abnormalities in sperm nuclei and chromatin can interfere with normal fertilization, embryonic development, implantation, and pregnancy. We aimed to study the impact of *H2BFWT* gene variants in sperm DNA on ICSI outcomes in couples undergoing ART treatment. One hundred and nineteen partners were divided into pregnant (G1) and non-pregnant (G2) groups. After semen analysis, complete DNA was extracted from purified sperm samples. The sequence of the *H2BFWT* gene was amplified by PCR and then subjected to Sanger sequencing. The results showed that there are three mutations in this gene: rs7885967, rs553509, and rs578953. Significant differences were shown in the distribution of alternative and reference alleles between G1 and G2 (*p* = 0.0004 and *p* = 0.0020, respectively) for rs553509 and rs578953. However, there was no association between these SNPs and the studied parameters. This study is the first to shed light on the connection between *H2BFWT* gene variants in sperm DNA and pregnancy after ICSI therapy. This is a pilot study, so further investigations about these gene variants at the transcriptional and translational levels will help to determine its functional consequences and to clarify the mechanism of how pregnancy can be affected by sperm DNA.

## 1. Introduction

Epigenetic and genetic defects in sperm DNA play an important role in male infertility problems and consequently in reproductive medicine. Intracytoplasmic sperm injection (ICSI) is a great solution for couples suffering from fertility problems, especially impaired sperm motility and count, but not sperm DNA and chromatin errors [1].

The epigenetic memory transferred by sperm during fertilization can aid epigenetic reprogramming of fertilized eggs and early embryo development by regulating gene expression patterns [2,3,4].

Any changes in this process may lead to reduced sperm capacity and reduced fertility [5,6]. In addition, sperm DNA abnormalities are clinically relevant as they are associated with a reduction in fertility potential [7,8]. Further, an abnormal histone–protamine ratio can lead to infertility [9,10]. Furthermore, changes in chromatin regulators or histone levels during spermatogenesis can lead to defects in development that can be transmitted from generation to generation [11,12].

Changes in epigenetic characteristics of sperm can subsequently be inherited by offspring and may affect their health [13,14,15,16].

The basic unit of chromatin is the nucleosome, a histone octameric protein complex that envelopes 147 bp of DNA. Each octamer contains two histone H2A-H2B dimers linked to two histone H3-H4 dimers [17,18].

During spermatogenesis, the chromatin is reorganized and testis-specific histones partially replace somatic histones [19]. This lead to an extremely compacted DNA in the sperm nucleus in comparison to somatic cell nuclei.

In a tightly controlled process, testis-specific histone variants such H2B histone family, member W, testis-specific protein (H2BFWT), the testis-specific histone H2B variant (TH2B) and the testis-specific histone H2A variant (TH2A) replace canonical histone [20,21].

Specific histone modifications are important, during the histone-to-protamine transition, to achieve a higher-order chromatin structure [22,23,24,25].

Chromatin structure is important for transcription, replication, DNA repair, and recombination, and thus relies on tight regulation [26].

During spermatogenesis, histone variants are expressed and regulate chromatin structure [27,28]. Furthermore, different types of histone modifications have been shown to have a role in promoting the conversion of histones to protamine, such as acetylation, ubiquitination, phosphorylation, and methylation [29]. These modifications are thought to weaken DNA–histone interactions, resulting in removing and replacing histones first by testing specific histone variants, then by transition proteins, and later by protamines [23,30,31].

In many species, histones are removed and DNA is condensed into highly compact nuclear protamine complexes by highly positively charged protamine. Therefore, the main characteristic of mature spermatozoa is highly condensed, inactive chromatin [32,33].

Gatewood et al. found that 15% of the human genome has histone conservation [34,35]. Another study reported only 3% to 5% [36,37]. Other studies showed that 10–15% of human genome and 1–10% of mice couples have histone-specific nucleosomes [38,39]. Furthermore, more than 10% of the genome in mature male sperm contains nucleosomes [40].

In recent years, the nucleosome retention phenomenon in the sperm genome has sparked debate, and various questions were raised concerning the functional influence of sperm nucleosomes. Nevertheless, the exact function and mechanism of histone retention remain unclear.

In comparison to their canonical counterparts, histone variants exhibit different functional characteristics, highlighted by their unique structure, expression patterns, and/or localization [41].

These retained histones are mainly located on gene promoters and regulatory elements with elevated content of unmethylated CpG regions, suggesting that they play a part in the transcriptional regulation and genome organization of these genes after oocyte fertilization [38,42]. In addition, a large portion of the histone-associated sperm genome is repetitive in nature [11].

TH2B largely replaces H2B during meiosis and remain the predominant type of this histone in round and elongated spermatids [43], suggesting that TH2B is important for meiotic and post-meiotic changes in cells [20,21].

There is a 95% sequence homology in the C-terminal region between TSH2B and somatic H2B, and there is a 70% sequence homology in the C-terminal region between H2BFWT and somatic H2B [44,45].

The gene-encoding H2B histone family W member, testis-specific (*H2BFWT*) protein is located on chromosome Xq22.2, and its amino acid sequence has a 45% homology with the amino acid sequence of somatic H2B. H2BFWT is expressed in sperm nuclei; previous studies have shown that H2BFWT protein colocalizes with telomeric sequences [45].

Studies investigating the association between *H2BFWT* gene variants and male infertility have brought valuable insights into the molecular mechanisms of sperm development and function. The study by Lee et al. (2009) identified a single nucleotide polymorphism (−9C>T) in the 5′ untranslated region (5′UTR) of the *H2BFWT* gene associated with male infertility [46]. They showed that this variation was significantly associated with sperm count, vitality, and non-azoospermic men. They showed that in vivo expression of H2BFWT in spermatozoa was dependent on the non-azoospermic −9C>T genotype [46]. The same results were observed in Chinese populations [47] and Iranian populations [48].

In a recent study, it was suggested that H2BFWT may be required to regulate spermatogenesis-related gene expression by reducing transcriptional barriers leading to infertility [49]. However, comparative results showed no correlation between male infertility and two variants in the *H2BFWT* gene: rs553509 and rs7885967 [50].

In a previous study, Amor et al. (2022) found no significant difference in allele frequency between heavy smokers and non-smokers for three homozygous SNPs in *H2BFWT*. Furthermore, the identified SNPs had no effect on sperm parameters and its DNA integrity [51].

Despite the understanding of chromatin condensation into protamine toroids in spermatozoa [52], DNA methylation, and genome-wide histone retention [36,40,53], the epigenetic factors of infertility and their impact on embryogenesis and pregnancy are still to be elucidated.

Therefore, the purpose of this study was to determine the relationship between the *H2BFWT* gene variations in spermatozoa DNA in male partners of couples undergoing assisted reproductive technology (ART) treatment and ICSI outcome (pregnancy).

## 2. Results

### 2.1. Studied Parameters among Studied Groups

One hundred and nineteen samples were divided into two groups according to pregnancy status as indicated in Table 1. Fourty-nine samples were obtained from pregnant couples “as controls” and seventy samples were obtained from non-pregnant couples “as cases”. The results of this study showed that only sperm count was significantly different between the pregnant and non-pregnant groups (55.72 vs. 80.08 × 10^6^/mL, *p* = 0.0152) (Table 1).

In the pregnant group, the number of fertilized eggs, percentage of fertilization, and number of cleaved eggs were significantly higher than the non-pregnant group (8.46 vs. 6.66, *p* = 0.0275; 81.68% vs. 72.79%, *p* = 0.0203 and 8.02 vs. 6.25, *p* = 0.0367) (Table 2).

### 2.2. Variant Calling and Quality Control

To determine the allele frequency of the *H2BFWT* gene, chromatographic data files (.ab1) were used to identify primary and secondary sequences using the Tracy tool (https://github.com/gear-genomics/tracy (accessed on 27 March 2023). FASTA data were mapped to the hg19 reference genome using the aligner bwa [54]. A BAM file containing 4 reads for each participant was created after performing forward and reverse Sanger reads for each participant. A list of possible SNP points was then generated. For the identification of all regions in the aligned data set where at least one read contains an allele that is different from the reference sequence, samtools mpileup was used [55]. All SNP variants were genotyped across all participants using WhatsHap [56,57].

All loci with 5% or greater allele frequency in all study participants were selected from all variations generated in the previous step. Three SNPs are located on the X chromosome. In fact, our genotyping algorithm genotyped the different variations located on the X chromosome in all individuals as 0/0 or 1/1. Then, Fisher’s exact test was used for Hardy–Weinberg equilibrium to test them. These variations did not deviate significantly from HWE. Therefore, our final SNP call set contained three SNPs. The corresponding positions and alleles of these variations are listed in Table 3 (relative to the reference sequence hg19).

Further, we note that the 1000 Genomes Project [58] has reported our variation calls. An additional quality control was conducted, in order to check the allelic distribution determined by these SNPs against the allelic distribution reported by 1000 Genomes and found that they matched well.

### 2.3. H2BFWT SNPs Distribution between Pregnant and Non-Pregnant

A table was created for each SNP in each group by calculating the reference and alternative allele counts for each group of participants. We aimed to evaluate the relationship between SNP alleles and each category. Fisher’s exact test was then used to compare the two groups regarding allele frequencies, and Benjamini–Hochberg correction (alpha = 0.05) was used for correction of multiple experiments. Two of the SNPs were found to be significant. All examined SNPs and their allele frequencies are summarized in Table 4.

*H2BFWT* is located on the X chromosome (q22.2 band). All our participants were male and thus SNPs rs578953, rs553509, and rs7885967 were found to be homozygous.

The rs553509 polymorphism is a missense variant that replaces arginine with histidine (Arg/His) in exon 1 (Table 4). The C and T allele frequencies at this locus were 16% and 84%, respectively, in pregnant couples, 38% and 62%, respectively, in non-pregnant couples. This variant, which differed significantly between pregnant and non-pregnant women (*p* = 0.0004), was predicted to be benign according to the Poly Phenyl-2 software (score 0.016, sensitivity 0.95; specificity 0.79).

rs578953 is an upstream gene polymorphism. This variant showed a significant difference in the distribution of the alternative and reference alleles between pregnant and non-pregnant women (*p* = 0.0020). The G and A allele frequencies at this locus were 86% and 14%, respectively, in pregnant couples and 97% and 3%, respectively, in non-pregnant couples (Table 4). However, the third variant, rs7885967, located in the 5′ untranslated region showed no significant difference (*p* = 0.1428) (Table 4).

### 2.4. Relationship between SNPs and Conventional Sperm Parameters and Clinical Outcomes following ICSI Treatment

To reveal the relationship between SNPs and sperm parameters and clinical outcomes after ICSI treatment, Wilcoxon rank-sum tests were done for each integration of SNPs and phenotypes. The purpose was to study whether the frequency of phenotypic values is not different in populations with various genotypes.

First, the frequency of genotype 0/0 was compared to the frequency of subjects with genotype 1/1. Then Benjamini–Hochberg correction was used again to correct for multiple tests (alpha = 0.05). There was no evidence of a correlation between these variations and the examined parameters.

## 3. Discussion

Sperm is the carrier that transports the paternal genome to the egg. For normal embryonic development, complete and intact genetic material is required. During spermatogenesis, the formation of spermatozoa leads to a sperm DNA nuclei that is extremely compact compared to somatic cell nuclei [59].

Several reports indicate that male gametes confer various epigenetic marks, RNA, and protein molecules on the fertilized egg. These factors play a crucial role in embryonic development and the future health of the offspring [33,60,61,62]. In addition, modified epigenetic signatures in sperm can be transferred to the next generation, which can affect their health [13,14,15,16].

However, various factors, such as nutritional status, lifestyle changes, dietary exposure, or environmental toxins such as alcohol abuse and smoking can influence the sperm epigenome [13,63,64,65].

DNA abnormalities are associated with abnormalities in chromatin packaging. Implantation failure of embryos derived from healthy oocyte is thought to be caused by sperm DNA damage [66]. Furthermore, there is abundant evidence that changes in sperm chromatin compaction during spermatogenesis are associated with fluctuations in the decondensation of chromatin in the ooplasm, which in turn may affect early development of the embryo [34,67].

In addition, chromatin decondensation is the first obvious change after the sperm enters the egg plasm, and is also a prerequisite for pronucleus formation. Therefore, nuclear decondensation can be used to evaluate the ability of sperm to fertilize [19,68].

However, there are few data on the relationship between condensation and decondensation of sperm chromatin and ongoing pregnancy [69,70,71].

The core histones in mature human sperm chromatin are largely replaced by protamine, thereby eliminating most chromatin patterns. However, these histones are not completely replaced during spermatogenesis and constitute approximately 15% of the essential chromosomal proteins in mature sperm [66,72]. Furthermore, a small percentage of histones remain associated with the sperm chromatin [36,53].

H3, H4, H2B, H2A, and histone isoform variants exist in human sperm, with the predominant histone being H2B [72]. *H2BFWT* and hTSH2B, two variants of H2B, have been cloned and characterized [44,45]. Histone variants are important for the process of eviction as they are able to promote the relaxation of the nucleosome structure and the interaction interfaces needed for the assembly of non-histone structural proteins on DNA [30,73].

Importantly, reductions in testis-specific histones can lead to abnormal sperm morphology, leading to male subfertility [74,75].

Epigenomic packaging changes in mice and humans suggest that paternal histones play a crucial role in the development of early embryos [36,40,53]. Current studies focus on determining whether aberrant epigenetics in gametes may lead to failure of embryogenesis and result in infertility [76].

Changes in epigenetic characteristics in sperm can be inherited by offspring and may affect the health of the offspring [13,14,15,16].

Imprinted gene regions such as promoters of discrete developmental transcription and signaling factors, homeobox (HOX) genes, and microRNAs are rich in these genetic markers [36].

Examining the relationship between *H2BFWT* gene mutations in human sperm DNA and their impact on ART outcomes provides valuable insights into the genetic factors that influence reproductive success. Although studies directly examining the impact of this specific gene on pregnancy and ART outcomes are limited, understanding the broader context of genetic variation in sperm DNA and its association with reproductive outcomes can help reveal potential effects. The use of polymorphic markers is not decisive, but it may associate with or contribute to the infertility condition. In addition, genetic screening for *H2BFWT* gene mutations or polymorphisms may help diagnose the underlying causes of infertility and guide personalized treatment strategies. Thus, we aimed in this study to investigate the clinical implications of the *H2BFWT* gene in couples undergoing assisted reproductive technologies (ART) therapy. *H2BFWT* is expressed in sperm nuclei and the expression level is related to the single nucleotide polymorphism in the 5′ untranslated region (5′UTR) that showed an association with male infertility [46]. Thus, the detected SNPs (rs553509 and rs578953) on the *H2BFWT* gene in this study showed a significant association with pregnancy after ICSI treatment, which can be attributed to the functional rule of these variants in gene expression levels. For instance, previous studies showed that some variations were significantly associated with sperm count, vitality, and non-azoospermic men as well as in vivo expression of *H2BFWT* in spermatozoa [46,47,48]. This was attributed to the crucial role of H2BFWT in the regulation of spermatogenesis leading to infertility [49]. Therefore, more functional studies are required to understand the molecular impact of the *H2BFWT* gene variants in the spermatogenesis process, which will explain the effect of their polymorphisms on the ICSI outcomes. However, the detected polymorphic variants may suggest their impact on the function/expression level of the *H2BFWT* gene and consequently affect the spermatogenesis processes. The clinical implications of these are highly important for ICSI outcomes, and the genetic markers can be considered before ICSI injection.

In the present study, we determined the variations in the *H2BFWT* gene in sperm DNA from men of couples undergoing ICSI treatment. Then, we investigated the possible role of these SNPs on pregnancy in order to clarify its basic correlation with male infertility. This is the first study, to the best of our knowledge, to examine the potential relationship between gene alterations in the *H2BFWT* gene and ICSI outcomes in couples undergoing ICSI treatment. Undoubtedly, successful reproduction involves not only a functional spermatozoon but also a functional oocyte as well. Thus, we cannot expect to predict ART outcomes by looking at sperm functionality alone. Nevertheless, developing novel sperm function assays should help us pinpoint defects of sperm function and, in turn, rule out their contribution to pregnancy failure.

In the pregnant group, the number of fertilized eggs, percentage of fertilization, and number of cleaved eggs were significantly higher than the non-pregnant group (*p* < 0.05) (Table 2).

The results of sequencing showed that three variants were identified: rs7885967, rs553509, and rs578953 (Table 4). The rs553509 polymorphism is a missense variant that replaces arginine with histidine (Arg/His) in Exon 1. The rs578953 is an upstream gene polymorphism. Both variants showed a significant difference of alternative and reference allele distributions between the pregnant and non-pregnant groups (*p* = 0. 0004 and *p* = 0.0020 respectively). The third variant showed no significant difference (*p* = 0.1428) (Table 4). However, there was no association between any of the SNPs and the studied parameters.

Male subfertility is actually associated with defects in sperm chromatin condensation. Pregnancy rates are higher when sperm exhibit normal chromatin compaction, and a greater count of sperm with normal chromatin condensation is related with increased early embryo cleavage rates [77,78].

Galotto et al., 2019 found that in fast and slow decondensers, the fertilization and cleavage rates were similar, but in fast decondensers, embryo quality was better. The results of this study suggest possible delayed negative effects on embryonic development [79].

Controversy remains in the literature regarding the importance of the decondensation process for the success of ART. Although some studies claim that ICSI results are not affected by chromatin compaction [80]. Others have pointed out that IUI results are also influenced by the ability of sperm to decondense [78].

Undoubtedly, we should keep in mind that fertilization requires a functional spermatozoon and a good quality oocyte. Therefore, sperm quality is not the only parameter to predict ART outcomes. However, the understanding of sperm function and the development of novel assays can facilitate addressing abnormalities in sperm function and their role in pregnancy failure.

*H2BFWT* has two SNPs associated with infertility. An SNP in the 5′UTR region (−9C>T) introduces a new start codon ATG at position −10 and causes a frameshift. Several studies confirmed the inability to produce H2BFWT protein [46,47,48].

This SNP has also been reported to be strongly associated with azoospermia [47]. Therefore, there is evidence that *H2BFWT* has an important function in spermatogenesis. The SNP at 368A>G, which replaces the amino acid at position 100 of histidine (H) with arginine (R) (H2BFWTH100R), is associated with a less severe oligozoospermia phenotype in which sperm is produced but in low numbers [49]. However, statistical analysis in this study showed that this SNP was not correlated with male infertility (*p* > 0.05).

## 4. Material and Methods

### 4.1. Subjects

Semen samples from 119 males who were admitted randomly to the Saarland University medical faculty, department of Obstetrics & Gynaecology, IVF Laboratory were enrolled in this prospective study. The inclusion criteria were as follows: the patients were healthy and phenotypically normal; their ethnic descents were categorized as European. Potential confounders, including body mass index, cigarette smoking, alcohol and caffeine use and those with an appreciable effect on the ICSI outcome were not retained in the study design model. All the patients included in this study were of fertility age and diagnosed with male factor infertility. Each male partner was subjected to an examination and each one who had genetic abnormalities was also excluded. 

The study was conducted in accordance with the Declaration of Helsinki and the study protocol was reviewed and approved by the Institutional Review Board of Saarland university (No. 195/11). Informed consent was obtained from all subjects involved in the study. 

Samples were obtained by masturbation. After liquefaction at 37 °C for 30 min, samples were evaluated according to the laboratory guideline of the World Health Organization (WHO) [81]. Patients were later divided into the pregnant patients group (*n* = 49) and the non-pregnant patients group (*n* = 70). 

### 4.2. Semen Analysis and Preparation for ICSI

Semen samples were fractionated by PureSperm media (40% and 80% gradient) (NidaCon International AB, Mölndal, Sweden). Then, the pellet was kept and washed. Supernatant was discarded, and the pellet was layered with G-IVF Plus medium (Vitrolife, Göteborg, Sweden) and kept at 37 °C for 1 h in the CO_2_ incubator. Finally, the supernatant was collected, and used for ICSI later.

### 4.3. Intracytoplasmic Sperm Injection Technique (ICSI)

After two hours, the retrieved oocytes were denudated using a hyaluronidase enzyme (Gynemed, Sierksdorf, Germany) and glass pipettes (Vitromed, Jena, Germany). Then, only the metaphase II oocytes were injected.

### 4.4. Identification of Genetic Variant in H2BFWT Gene

Isolate II RNA/DNA/Protein (Phenol-free) Kit (Bioline, London, UK) was used to extract genomic DNA from purified semen specimens. Finally, the extracted DNA’s quantity and purity was evaluated using Nanodrop spectrophotometer ND-2000c (Thermo Scientific, Waltham, MA, USA) and kept at −80 °C. The *H2BFWT* gene was amplified using a conventional PCR method. Primer3 was used to design the primers (F: forward and R: reverse) depending on the reference sequencing for the three genes retrieved from GenBank [82].

The polymerase chain reaction (PCR) was performed using MyTaq^TM^HS Red Mix Kit (Bioline, London, UK) in a 30 µL mixture as follows: 0.6 µL of Primers (20 µM each) (*F:* tggcatggatcagctgagaa and *R:* ggacactccctaagcctact) was added to 20 ng/µL of DNA template and 15 µL of 2× MyTaqHS Red Mix. Then, nuclease-free water was added to the previous mixture up to 30 µL.

The thermocycler program was applied to identify the *H2BFWT* gene variants using C1000^TM^ Thermal cycler (Bio-Rad Laboratories GmbH, Feldkirchen, Germany) (annealing temperature of 64 °C;). Then, PCR products were purified using Qiagen Miniprep PCR-purification HT and sequenced utilizing the Sanger sequence method and two single Read HT were constructed for all genes (Qiagen, Hilden, Germany).

### 4.5. Statistical Analysis

IBM SPSS for Windows software package version 24.0 (IBM SPSS, Chicago, IL, USA) was used to analyze the data. The samples were non-normally distributed according to Shapiro test, skewness test, z-score, and kurtosis test. Mann–Whitney U test was used to compare study parameters between pregnant and non-pregnant groups, and Spearman analysis was performed to determine the association between different study parameters.

The allele frequency of each gene was determined using the Tracy tool (https://github.com/gear-genomics/tracy (accessed on 27 March 2023)). Variants were called using samtools mpileup [55], aligner bwa [54], and WhatsHap [56,57]. Then, regions with an allelic distribution more than 5% among all study participants were chosen to be evaluated for Hardy–Weinberg Equilibrium using Fisher’s exact test. Additionally, Fisher’s exact test was used to determine significant associations of allele frequencies between pregnant and non-pregnant groups, with correction for multiple testing using Benjamini–Hochberg correction (alpha = 0.05).

## 5. Conclusions

In conclusion, this pilot study demonstrated that two important SNP positions (rs553509 and rs578953) on the *H2BFWT* gene were associated with pregnancy after ICSI treatment. However, these results must be validated in a larger patient population. Therefore, further studies of these genetic variants at the transcriptional and translational levels are needed to determine the functional consequences of the identified variants and to determine the mechanisms of how sperm DNA affects fertilization rates, particularly during the early stages of embryonic development. These findings add further evidence to the importance of genomic research studies to investigate the genetic causes of male infertility.

Taken together, by understanding the role of SNPs in sperm DNA, we can imagine a future where personalized medicine and targeted interventions will revolutionize the field of reproductive health and ultimately improve the well-being of parents and offspring.

## Figures and Tables

**Table 1:** Comparison of standard semen parameters between the pregnant group (*n* = 49) and the non-pregnant group (*n* = 70).

Parameters	Pregnant (*n* = 49)	Non-Pregnant (*n* = 70)	*p*-Value
Semen volume (mL)	3.16	3.28	0.6642
Sperm count (10^6^ per mL)	55.72	80.08	0.0152 *
Total motility (%)	36.76	40.08	0.3643
Progressive motility (PR) (%)	13.12	16.27	0.1120
Normal form (%)	3.60	4.40	0.1173

Note: Results expressed as median values. * *p*-value is statistically significant at the 0.05 level.

**Table 2:** Comparison of intracytoplasmic sperm injection outcomes between the pregnant group (*n* = 49) and the non-pregnant group (*n* = 70).

Parameters	Pregnant (*n* = 49)	Non-Pregnant (*n* = 70)	*p*-Value
Number of Collected egg	13.74	12	0.1590
Number of injected eggs	10.28	8.79	0.1402
Number of fertilised egg	8.46	6.55	0.0275 *
% of fertilized egg	81.68	72.79	0.0203 *
Number of Cleaved eggs	8.02	6.25	0.0367 *

Note: Results expressed as median values. * *p*-value is statistically significant at the 0.05 level.

**Table 3:** Allele frequencies of the detected SNPs across all samples.

Genomic Position (hg19)		ID	Reference Allele	Alternative Allele	Allele Frequency
Chromosome X	103268333	rs578953	G	A	0.09
Chromosome X	103267865	rs553509	C	T	0.72
Chromosome X	103268241	rs7885967	G	A	0.60

**Table 4:** Summary of results obtained by direct sequencing of the *H2BFWT* gene.

SNP	Gene Region	AA Change	ID	Pregnant		Non-Pregnant		*p*-Value
				Genotype	Allele	Genotype	Allele	
chrX 103268333G>A	Upstream	N/A	rs578953	A/A (7) 0.14 G/A (0) 0.0 G/G (42) 0.86	A = 0.14 G = 0.86	A/A (2) 0.03 G/A (0) 0.0 G/G (68) 0.97	A = 0.03 G = 0.97	0.0020 **
chrX 103267865C>T	Exon 1	R/H	rs553509	T/T (41) 0.84 C/T (0) 0.0 C/C (8) 0.16	T = 0.84 C = 0.16	T/T (43) 0.62 C/T (0) 0.0 C/C (26) 0.38	T = 0.62 C = 0.38	0.0004 **
chrX 103268241G>A	5 prime UTR	N/A	rs7885967	A/A (32) 0.65 G/A (0) 0.0 G/G (17) 0.35	A = 0.65 G = 0.35	A/A (39) 0.56 G/A (0) 0.0 G/G (31) 0.44	A = 0.56 G = 0.44	0.1428

UTR: Untranslated region, N/A: Not applicable. ** *p*-value is highly statistically significant at the 0.01 level.

## Data Availability

The data presented in this study are available on request from the corresponding author. The data are not publicly available due to ethical restrictions.

## References

[1] Vavouri T., Lehner B. (2011). Chromatin Organization in Sperm May Be the Major Functional Consequence of Base Composition Variation in the Human Genome. PLoS Genet..

[2] Van Der Heijden G.W., Ramos L., Baart E.B., Van Den Berg I.M., Derijck A.A.H.A., Van Der Vlag J., Martini E., De Boer P. (2008). Sperm-Derived Histones Contribute to Zygotic Chromatin in Humans. BMC Dev. Biol..

[3] Paradowska A.S., Miller D., Spiess A.N., Vieweg M., Cerna M., Dvorakova-Hortova K., Bartkuhn M., Schuppe H.C., Weidner W., Steger K. (2012). Genome Wide Identification of Promoter Binding Sites for H4K12ac in Human Sperm and Its Relevance for Early Embryonic Development. Epigenetics.

[4] Van De Werken C., Van Der Heijden G.W., Eleveld C., Teeuwssen M., Albert M., Baarends W.M., Laven J.S.E., Peters A.H.F.M., Baart E.B. (2014). Paternal Heterochromatin Formation in Human Embryos Is H3K9/HP1 Directed and Primed by Sperm-Derived Histone Modifications. Nat. Commun..

[5] Peters A.H.F.M., Mermoud J.E., O’carroll D., Pagani M., Schweizer D., Brockdorff N., Jenuwein T. (2001). Histone H3 Lysine 9 Methylation Is an Epigenetic Imprint of Facultative Heterochromatin. Nat. Genet..

[6] Schon S.B., Luense L.J., Wang X., Bartolomei M.S., Coutifaris C., Garcia B.A., Berger S.L. (2019). Histone Modification Signatures in Human Sperm Distinguish Clinical Abnormalities. J. Assist. Reprod. Genet..

[7] Erenpreiss J., Spano M., Erenpreisa J., Bungum M., Giwercman A. (2006). Sperm Chromatin Structure and Male Fertility: Biological and Clinical Aspects. Asian J. Androl..

[8] Emery B.R., Carrell D.T. (2006). The Effect of Epigenetic Sperm Abnormalities on Early Embryogenesis. Asian J. Androl..

[9] Aoki V.W., Emery B.R., Liu L., Carrell D.T. (2006). Protamine Levels Vary between Individual Sperm Cells of Infertile Human Males and Correlate with Viability and DNA Integrity. J. Androl..

[10] Hamad M.F., Shelko N., Kartarius S., Montenarh M., Hammadeh M.E. (2014). Impact of Cigarette Smoking on Histone (H2B) to Protamine Ratio in Human Spermatozoa and Its Relation to Sperm Parameters. Andrology.

[11] Ihara M., Meyer-Ficca M.L., Leu N.A., Rao S., Li F., Gregory B.D., Zalenskaya I.A., Schultz R.M., Meyer R.G. (2014). Paternal Poly (ADP-Ribose) Metabolism Modulates Retention of Inheritable Sperm Histones and Early Embryonic Gene Expression. PLoS Genet..

[12] Siklenka K., Erkek S., Godmann M., Lambrot R., McGraw S., Lafleur C., Cohen T., Xia J., Suderman M., Hallett M. (2015). Disruption of Histone Methylation in Developing Sperm Impairs Offspring Health Transgenerationally. Science.

[13] Schaefer S., Nadeau J.H. (2015). The Genetics of Epigenetic Inheritance: Modes, Molecules, and Mechanisms. Q. Rev. Biol..

[14] Champroux A., Cocquet J., Henry-Berger J., Drevet J.R., Kocer A. (2018). A Decade of Exploring the Mammalian Sperm Epigenome: Paternal Epigenetic and Transgenerational Inheritance. Front. Cell Dev. Biol..

[15] Xu X., Miao Z., Sun M., Wan B. (2021). Epigenetic Mechanisms of Paternal Stress in Offspring Development and Diseases. Int. J. Genom..

[16] Fitz-James M.H., Cavalli G. (2022). Molecular Mechanisms of Transgenerational Epigenetic Inheritance. Nat. Rev. Genet..

[17] Luger K., Mäder A.W., Richmond R.K., Sargent D.F., Richmond T.J. (1997). Crystal Structure of the Nucleosome Core Particle at 2.8 Å Resolution. Nature.

[18] McGuffee S.R., Smith D.J., Whitehouse I. (2013). Quantitative, Genome-Wide Analysis of Eukaryotic Replication Initiation and Termination. Mol. Cell.

[19] Herman Van Roijen J., Ooms M.P., Spaargaren M.C., Baarends W.M., Weber R.F.A., Anton Grootegoed J., Vreeburg J.T.M. (1998). Immunoexpression of Testis-Specific Histone 2B in Human Spermatozoa and Testis Tissue. Hum. Reprod..

[20] Rathke C., Baarends W.M., Awe S., Renkawitz-Pohl R. (2014). Chromatin Dynamics during Spermiogenesis. Biochim. Biophys. Acta—Gene Regul. Mech..

[21] Ausio J., Zhang Y., Ishibashi T. (2016). Histone Variants and Posttranslational Modifications in Spermatogenesis and Infertility. Epigenetic Biomarkers and Diagnostics.

[22] Bošković A., Torres-Padilla M.E. (2013). How Mammals Pack Their Sperm: A Variant Matter. Genes Dev..

[23] Bao J., Bedford M.T. (2016). Epigenetic Regulation of the Histone-to-Protamine Transition during Spermiogenesis. Reproduction.

[24] Hada M., Masuda K., Yamaguchi K., Shirahige K., Okada Y. (2017). Identification of a Variant-Specific Phosphorylation of TH2A during Spermiogenesis. Sci. Rep..

[25] Hao S.L., Da Ni F., Yang W.X. (2019). The Dynamics and Regulation of Chromatin Remodeling during Spermiogenesis. Gene.

[26] Kornberg R.D., Lorch Y. (2020). Primary Role of the Nucleosome. Mol. Cell.

[27] McCarrey J.R., Geyer C.B., Yoshioka H. (2005). Epigenetic Regulation of Testis-Specific Gene Expression. Ann. N. Y. Acad. Sci..

[28] Govin J., Escoffier E., Rousseaux S., Kuhn L., Ferro M., Thévenon J., Catena R., Davidson I., Garin J., Khochbin S. (2007). Pericentric Heterochromatin Reprogramming by New Histone Variants during Mouse Spermiogenesis. J. Cell Biol..

[29] Luense L.J., Wang X., Schon S.B., Weller A.H., Lin Shiao E., Bryant J.M., Bartolomei M.S., Coutifaris C., Garcia B.A., Berger S.L. (2016). Comprehensive Analysis of Histone Post-Translational Modifications in Mouse and Human Male Germ Cells. Epigenetics Chromatin.

[30] Barral S., Morozumi Y., Tanaka H., Montellier E., Govin J., de Dieuleveult M., Charbonnier G., Couté Y., Puthier D., Buchou T. (2017). Histone Variant H2A.L.2 Guides Transition Protein-Dependent Protamine Assembly in Male Germ Cells. Mol. Cell.

[31] Hoghoughi N., Barral S., Vargas A., Rousseaux S., Khochbin S. (2018). Histone Variants: Essential Actors in Male Genome Programming. J. Biochem..

[32] Balhorn R. (2007). The Protamine Family of Sperm Nuclear Proteins. Genome Biol..

[33] Castillo J., Estanyol J.M., Ballescà J.L., Oliva R. (2015). Human Sperm Chromatin Epigenetic Potential: Genomics, Proteomics, and Male Infertility. Asian J. Androl..

[34] Gatewood J.M., Cook G.R., Balhorn R., Bradbury E.M., Schmid C.W. (1987). Sequence-Specific Packaging of DNA in Human Sperm Chromatin. Science.

[35] Oliva R. (2006). Protamines and Male Infertility. Hum. Reprod. Update.

[36] Hammoud S.S., Nix D.A., Zhang H., Purwar J., Carrell D.T., Cairns B.R. (2009). Distinctive Chromatin in Human Sperm Packages Genes for Embryo Development. Nature.

[37] Carrell D.T., Hammoud S.S. (2010). The Human Sperm Epigenome and Its Potential Role in Embryonic Development. Mol. Hum. Reprod..

[38] Erkek S., Hisano M., Liang C.Y., Gill M., Murr R., Dieker J., Schübeler D., Van Der Vlag J., Stadler M.B., Peters A.H.F.M. (2013). Molecular Determinants of Nucleosome Retention at CpG-Rich Sequences in Mouse Spermatozoa. Nat. Struct. Mol. Biol..

[39] Yamaguchi K., Hada M., Fukuda Y., Inoue E., Makino Y., Katou Y., Shirahige K., Okada Y. (2018). Re-Evaluating the Localization of Sperm-Retained Histones Revealed the Modification-Dependent Accumulation in Specific Genome Regions. Cell Rep..

[40] Brykczynska U., Hisano M., Erkek S., Ramos L., Oakeley E.J., Roloff T.C., Beisel C., Schübeler D., Stadler M.B., Peters A.H.F.M. (2010). Repressive and Active Histone Methylation Mark Distinct Promoters in Human and Mouse Spermatozoa. Nat. Struct. Mol. Biol..

[41] Martire S., Banaszynski L.A. (2020). The Roles of Histone Variants in Fine-Tuning Chromatin Organization and Function. Nat. Rev. Mol. Cell Biol..

[42] Jung Y.H., Sauria M.E.G., Lyu X., Cheema M.S., Ausio J., Taylor J., Corces V.G. (2017). Chromatin States in Mouse Sperm Correlate with Embryonic and Adult Regulatory Landscapes. Cell Rep..

[43] Montellier E., Boussouar F., Rousseaux S., Zhang K., Buchou T., Fenaille F., Shiota H., Debernardi A., Héry P., Curtet S. (2013). Chromatin-to-Nucleoprotamine Transition Is Controlled by the Histone H2B Variant TH2B. Genes Dev..

[44] Zalensky A.O., Siino J.S., Gineitis A.A., Zalenskaya I.A., Tomilin N.V., Yau P., Morton Bradbury E. (2002). Human Testis/Sperm-Specific Histone H2B (HTSH2B) molecular cloning and characterization. J. Biol. Chem..

[45] Churikov D., Siino J., Svetlova M., Zhang K., Gineitis A., Morton Bradbury E., Zalensky A. (2004). Novel Human Testis-Specific Histone H2B Encoded by the Interrupted Gene on the X Chromosome. Genomics.

[46] Lee J., Park H.S., Kim H.H., Yun Y.J., Lee D.R., Lee S. (2009). Functional Polymorphism in H2BFWT-5′UTR Is Associated with Susceptibility to Male Infertility. J. Cell. Mol. Med..

[47] Ying H.Q., Scott M.B., Zhou-Cun A. (2012). Relationship of SNP of H2BFWT Gene to Male Infertility in a Chinese Population with Idiopathic Spermatogenesis Impairment. Biomarkers.

[48] Rafatmanesh A., Nikzad H., Ebrahimi A., Karimian M., Zamani T. (2018). Association of the c.-9C>T and c.368A>G Transitions in H2BFWT Gene with Male Infertility in an Iranian Population. Andrologia.

[49] Ding D., Pang M.Y.H., Deng M., Nguyen T.T., Sun X., Xu Z., Zhang Y., Zhai Y., Yan Y., Ishibashi T. (2022). Testis-Specific H2BFWT Disrupts Nucleosome Integrity through Reductions of DNA-Histone Interactions. bioRxiv.

[50] Haji Ebrahim Zargar H., Mohseni Meybodi A., Sabbaghian M., Shahhoseini M., Asadpor U., Sadighi Gilani M.A., Chehrazi M., Farhangniya M., Shahzadeh Fazeli S.A. (2015). Association of Two Polymorphisms in H2B.W Gene with Azoospermia and Severe Oligozoospermia in an Iranian Population. Int. J. Fertil. Steril..

[51] Amor H., Jankowski P.M., Dahadhah F.W., Al Zoubi M.S., Hammadeh M.E. (2022). Impact of Tobacco Smoking in Association with H2BFWT, PRM1 and PRM2 Genes Variants on Male Infertility. Andrologia.

[52] Miller D., Brinkworth M., Iles D. (2010). Paternal DNA Packaging in Spermatozoa: More than the Sum of Its Parts? DNA, Histones, Protamines and Epigenetics. Reproduction.

[53] Arpanahi A., Brinkworth M., Iles D., Krawetz S.A., Paradowska A., Platts A.E., Saida M., Steger K., Tedder P., Miller D. (2009). Endonuclease-Sensitive Regions of Human Spermatozoal Chromatin Are Highly Enriched in Promoter and CTCF Binding Sequences. Genome Res..

[54] Li H., Durbin R. (2009). Fast and Accurate Short Read Alignment with Burrows-Wheeler Transform. Bioinformatics.

[55] Li H., Handsaker B., Wysoker A., Fennell T., Ruan J., Homer N., Marth G., Abecasis G., Durbin R. (2009). The Sequence Alignment/Map Format and SAMtools. Bioinformatics.

[56] Ebler J., Haukness M., Pesout T., Marschall T., Paten B. (2018). Haplotype-Aware Genotyping from Noisy Long Reads. bioRxiv.

[57] Patterson M.D., Marschall T., Pisanti N., Van Iersel L., Stougie L., Klau G.W., Schönhuth A. (2015). WhatsHap: Weighted Haplotype Assembly for Future-Generation Sequencing Reads. J. Comput. Biol..

[58] Altshuler D.M., Durbin R.M., Abecasis G.R., Bentley D.R., Chakravarti A., Clark A.G., Donnelly P., Eichler E.E., Flicek P., Gabriel S.B. (2012). An Integrated Map of Genetic Variation from 1,092 Human Genomes. Nature.

[59] Marchiani S., Tamburrino L., Muratori M., Baldi E., Marchiani S., Muratori M. (2020). Spermatozoal Chromatin Structure: Role in Sperm Functions and Fertilization.

[60] Azpiazu R., Amaral A., Castillo J., Estanyol J.M., Guimerà M., Ballescà J.L., Balasch J., Oliva R. (2014). High-Throughput Sperm Differential Proteomics Suggests That Epigenetic Alterations Contribute to Failed Assisted Reproduction. Hum. Reprod..

[61] Chen L.Y., Willis W.D., Eddy E.M. (2016). Targeting the Gdnf Gene in Peritubular Myoid Cells Disrupts Undifferentiated Spermatogonial Cell Development. Proc. Natl. Acad. Sci. USA.

[62] Dimofski P., Meyre D., Dreumont N., Leininger-Muller B. (2021). Consequences of Paternal Nutrition on Offspring Health and Disease. Nutrients.

[63] Carone B.R., Fauquier L., Habib N., Shea J.M., Hart C.E., Li R., Bock C., Li C., Gu H., Zamore P.D. (2010). Paternally Induced Transgenerational Environmental Reprogramming of Metabolic Gene Expression in Mammals. Cell.

[64] Ng S.-F., Lin R.C.Y., Laybutt D.R., Barres R., Owens J.A., Morris M.J. (2010). Chronic High-Fat Diet in Fathers Programs b-Cell Dysfunction in Female Rat Offspring. Nature.

[65] Wei J., Sun X., Chen Y., Li Y., Song L., Zhou Z., Xu B., Lin Y., Xu S. (2014). Perinatal Exposure to Bisphenol A Exacerbates Nonalcoholic Steatohepatitis-like Phenotype in Male Rat Offspring Fed on a High-Fat Diet. J. Endocrinol..

[66] Gusse M., Sautière P., Bélaiche D., Martinage A., Roux C., Dadoune J.P., Chevaillier P. (1986). Purification and Characterization of Nuclear Basic Proteins of Human Sperm. Biochim. Biophys. Acta—Gen. Subj..

[67] Lewis J.D., Song Y., De Jong M.E., Bagha S.M., Ausió J. (2003). A Walk Though Vertebrate and Invertebrate Protamines. Chromosoma.

[68] Oliva R., Dixon G.H. (1991). Vertebrate Protamine Genes and the Histone-to-Protamine Replacement Reaction. Prog. Nucleic Acid Res. Mol. Biol..

[69] Hammadeh M.E., Strehler E., Zeginiadou T., Rosenbaum P., Schmidt W. (2001). Chromatin decondensation of human sperm in vitro and its relation to fertilization rate after ICSI. Arch. Androl..

[70] Tavalaee M., Razavi S., Nasr-Esfahani M.H. (2009). Influence of Sperm Chromatin Anomalies on Assisted Reproductive Technology Outcome. Fertil. Steril..

[71] Menezo Y., Clement P., Amar E. (2017). Evaluation of Sperm DNA Structure, Fragmentation and Decondensation: An Essential Tool in the Assessment of Male Infertility. Transl. Androl. Urol..

[72] Gatewood J.M., Cook G.R., Balhorn R., Schmid C.W., Bradbury E.M. (1990). Isolation of Four Core Histones from Human Sperm Chromatin Representing a Minor Subset of Somatic Histones. J. Biol. Chem..

[73] Tachiwana H., Kagawa W., Shiga T., Osakabe A., Miya Y., Saito K., Hayashi-Takanaka Y., Oda T., Sato M., Park S.Y. (2011). Crystal Structure of the Human Centromeric Nucleosome Containing CENP-A. Nature.

[74] Tanaka H., Iguchi N., Isotani A., Kitamura K., Toyama Y., Matsuoka Y., Onishi M., Masai K., Maekawa M., Toshimori K. (2005). HANP1/H1T2, a Novel Histone H1-like Protein Involved in Nuclear Formation and Sperm Fertility. Mol. Cell. Biol..

[75] Couldrey C., Carlton M.B.L., Nolan P.M., Colledge W.H., Evans M.J. (1999). A Retroviral Gene Trap Insertion into the Histone 3.3A Gene Causes Partial Neonatal Lethality, Stunted Growth, Neuromuscular Deficits and Male Sub-Fertility in Transgenic Mice. Hum. Mol. Genet..

[76] Tesarik J. (2005). Paternal Effects on Cell Division in the Human Preimplantation Embryo. Reprod. Biomed. Online.

[77] Depa-Martynów M., Kempisty B., Lianeri M., Jagodziński P.P., Jȩdrzejczak P. (2007). Association between Fertilin β, Protamines 1 and 2 and Spermatid-Specific Linker Histone H1-like Protein MRNA Levels, Fertilization Ability of Human Spermatozoa, and Quality of Preimplantation Embryos. Folia Histochem. Cytobiol..

[78] Irez T., Sahmay S., Ocal P., Goymen A., Senol H., Erol N., Kaleli S., Guralp O. (2015). Investigation of the Association between the Outcomes of Sperm Chromatin Condensation and Decondensation Tests, and Assisted Reproduction Techniques. Andrologia.

[79] Galotto C., Cambiasso M.Y., Julianelli V.L., Valzacchi G.J.R., Rolando R.N., Rodriguez M.L., Calvo L., Calvo J.C., Romanato M. (2019). Human Sperm Decondensation in Vitro Is Related to Cleavage Rate and Embryo Quality in IVF. J. Assist. Reprod. Genet..

[80] Karydis S., Asimakopoulos B., Papadopoulos N., Vakalopoulos I., Al-Hasani S., Nikolettos N. (2005). ICSI Outcome Is Not Associated with the Incidence of Spermatozoa with Abnormal Chromatin Condensation. In Vivo.

[81] World Health Organization (2010). WHO Laboratory Manual for the Examination and Processing of Human Semen.

[82] Untergasser A., Cutcutache I., Koressaar T., Ye J., Faircloth B.C., Remm M., Rozen S.G. (2012). Primer3-New Capabilities and Interfaces. Nucleic Acids Res..

